# Strain elastography imaging for early detection and prediction of tumor response to concurrent chemo-radiotherapy in locally advanced cervical cancer: feasibility study

**DOI:** 10.1186/s12885-017-3411-5

**Published:** 2017-06-19

**Authors:** Yan Xu, Lijing Zhu, Baorui Liu, Tong Ru, Huanhuan Wang, Jian He, Song Liu, Xiaofeng Yang, Zhengyang Zhou, Tian Liu

**Affiliations:** 1 Department of Radiology, Nanjing Drum Tower Hospital, The Affiliated Hospital of Nanjing University Medical School, Nanjing, 210008 China; 2 Department of Obstetrics and Gynecology, Nanjing Drum Tower Hospital, The Affiliated Hospital of Nanjing University Medical School, Nanjing, 210008 China; 3 The Comprehensive Cancer Centre of Nanjing Drum Tower Hospital, The Affiliated Hospital of Nanjing University Medical School, Nanjing, 210008 China; 4 Department of Radiation Oncology and Winship Cancer Institute, Emory University, Atlanta, GA 30322 USA

**Keywords:** Elastography, Tumor response, Cervical cancer, Concurrent chemo-radiotherapy

## Abstract

**Background:**

To investigate the feasibility of strain elastography imaging in early detecting and predicting treatment response in patients receiving concurrent chemo-radiotherapy (CCRT) for locally advanced cervical cancer.

**Methods:**

Between January 2015 and June 2016, 47 patients with locally advanced cervical cancer were enrolled in a feasibility study approved by the institutional review board. All patients underwent CCRT and received strain elastography examinations at 4 time points: pre-therapy (baseline), 1 week and 2 weeks during, as well as immediately post CCRT. Treatment response was evaluated by MRI at the time of diagnosis and immediately after CCRT. Based on the MRI findings, the treatment outcome was characterized as complete response (CR), partial response (PR), stable disease (SD) and progressive disease (PD). Strain ratio of the normal parametrial tissue vs. cervical tumor was calculated and compared with the clinical outcome.

**Results:**

Out of the 47 patients, 36 patients who completed all 4 examinations were included in the analyses: 25 were classified as CR, 11 as PR, and 0 in the SD/PD groups. Strain ratios were significantly different among the time points in both the CR group (*F* = 87.004, *p* < 0.001) and PR group (*F* = 38.317, *p* < 0.001). Strain ratios were significantly difference between the CR and PR groups (*F* = 7.203 *p* = 0.011). Strain ratios between the CR group and PR group were significantly different at 1 week after treatment initiation (*p* < 0.05). Compared to the baseline, a significant decrease in the CR group was observed at week 1, week 2 and post treatment (all *p* < 0.001), while a significant decrease in the PR group was shown in week 2 and post treatment (both *p* < 0.05), but not at week 1 during CCRT (*p* = 0.084).

**Conclusions:**

We have conducted a prospective longitudinal study to evaluate tumor response in women receiving CCRT for cervical cancers. This study has demonstrated the potential of strain elastography imaging in monitoring and early predicting tumor response induced by CCRT.

**Electronic supplementary material:**

The online version of this article (doi:10.1186/s12885-017-3411-5) contains supplementary material, which is available to authorized users.

## Background

Cervical cancer is the most common gynecological malignancies in the world, responsible for an estimation of 265,672 deaths in 2012, 88% of which occurred in the developing countries [1]. Currently, concurrent chemo-radiotherapy (CCRT) is considered the standard treatment option for patients with locally advanced cervical cancer. Studies have shown that CCRT improves overall and progression-free survival, as well as decreases local and distant recurrence, yet at the expense of varying degree of toxicity and morbidity [2–4]. In addition, due to tumor heterogeneity, it is unlikely that all cancers will respond equally to a specific treatment regimen [5]. Thus, successful treatment that leads to a better outcome in cervical cancer necessitates accurate clinical evaluation. In recent year, increasing efforts were devoted to the early detection and prognostic assessments of the treatment-associated change in tumor burden [6].

Several functional imaging techniques, such as diffusion-weighted (DW) magnetic resonance imaging (MRI), dynamic contrast-enhanced (DCE) MRI, 18 F–fluorodeoxyglucose-positron emission tomography (FDG-PET), have been introduced to evaluate the effect of chemo-radiotherapyin cervical cancer [7–9]. These functional imaging techniques have demonstrated superior capability to conventional imaging in terms of identifying biological or molecular changes which occur prior to visible or measureable change in tumor size [10]. However, most of these technologies are not used in routine surveillance due to increased radiation burden, potential contrast agent’s adverse reaction, long scan time and high cost, especially for patients who require long-term follow-ups. These limitations underline the need for a reliable, convenient and low-cost functional imaging biomarker for tumor response to treatment.

Sonoelastography is a well-established imaging modality that could provide the information of tissue elasticity (stiffness) that is complementary to the morphology and vascularity information provided by conventional sonographic examinations [11]. An important property of tissue is its intrinsic elasticity, which changes under the influence of pathologic processes, such as inflammation and neoplasm. Various groups have investigated sonoelastography in cancer detection and differential diagnosis in several organs, such as breast, liver, thyroid, and prostate [12–15]. In these cases, the malignant tissues are shown less compressible, resulting in less strain than the normal tissues under uniform stress. Recently, sonoelastography imaging was used to study the normal and abnormal cervix [16–19]. Several studies have shown the potential of sonoelastography in monitoring and predicting the therapeutic response such as the responding and non-responding malignant tissues in patients following CCRT [20, 21].

The purpose of this study is to investigate the utility of strain elastography in monitoring and early predicting therapy response to CCRT in patients with locally advanced cervical cancer. To the best of our knowledge, only one study with four cases had investigated the role of strain elastography as imaging biomarkers for prediction of therapeutic response in cervical cancers [22]. Therefore, our study aimed to investigate whether the strain ratio can be used as imaging biomarker in evaluating early response in 1 or 2 weeks after the initiation of treatment in patients with locally advanced cervical cancer.

## Methods

### Patients and tumor characteristics

This study was approved by our institutional review board, and written informed consents were obtained from all patients. Between January 2015 and June 2016, we prospectively enrolled 47 consecutive patients with histologically confirmed cervical cancer who were scheduled to receive CCRT at our hospital. Each patient was staged according to the criteria of the International Federation of Gynecology and Obstetrics (FIGO). The inclusion criteria were as follows: (1) age over 21, (2) FIGO Stage IB to IV, and (3) no history of chemotherapy or radiotherapy.

### CCRT treatment

All patients were treated with a combination of radiotherapy and chemotherapy. Radiotherapy consisted of external beam radiotherapy (EBRT) and intracavitary high-dose-rate brachytherapy (ICR). EBRT was delivered to the whole pelvis with a total dose of 50 Gy (a daily dose of 2 Gy and 5 times per week). EBRT was accompanied by concurrent chemotherapy, as follows: three-four cycles of every 2 weeks Nedaplatin (40-60 mg/m2) plus Paclitaxel (80 mg/m2) in 32 patients. ICR was initiated after an EBRT dose of 50 Gy. ICR was delivered twice a week with a dose of 5 Gy at point A (6 times, total dose 30Gy). The definition of point A follows the American Brachytherapy Society recommendation [23]. The entire CCRT for each patient was completed within 8 weeks.

### Treatment response evaluation

The treatment response of the CCRT was determined by the shrinkage of the longest diameter of the cervical cancer with MRI prior to and right after therapy completion. All MRI was performed with a 3.0-T MRI scanner (Achieva 3.0 T, Philips Healthcare, Best, the Netherlands) with a 16-channel torso phased-array body coil at the time of diagnosis and immediately after therapy completion. Two radiologists independently evaluated longest tumor diameter based on T2-weighted images according to the corresponding diffusion-weighted as well as contrast enhanced images with the maximal magnification and compared them in consensus. The change of tumor size was calculated according to the following equation: change in tumor size % = (pre-longest diameter-post-longest diameter)/pre-longest diameter × 100%. The clinical responses were classified using the Response Evaluation Criteria in Solid Tumors (RECIST) 1.1 criteria [24] in the following 4 categories: complete response (CR), partial response (PR), stable disease (SD) and progressive disease (PD). CR is defined as no residual cancer, PR as at least a 30% decrease in the sum of diameters of the cancer, PD as at least 20% increase and SD as no sufficient shrinkage to qualify for PR or sufficient increase to qualify for PD.

### Strain elastography imaging and analysis

All patients underwent ultrasound examinations at the following 4 time points: prior to CCRT, at week 1 and week 2 during CCRT, as well as within 1 week post CCRT. Ultrasound data were acquired using GE Voluson E8 ultrasound machine (GE Medical Systems, USA) with a 3D/4D endocavitary convex array transducer (GE RIC5–9, bandwidth 5–9 MHz). All the examinations were performed by a single ultrasonographer with seven-year experience, who was blinded to the treatment outcome and MRI results.

The ultrasound examination was performed with the patient in the lithotomy position (with an empty bladder). A disposable condom with coupling gel was used to cover the endocavitary probe which was gently inserted in the anterior vaginal fornix. All ultrasound data were acquired with the same settings: 5.0 cm depth, 1 focal zone, 7 Gy map, −15 gain, 121°angle, 3 persist, 2 enhance, 20 reject and 7 dynamic control. This setting was kept consistent throughout the study to ensure quantitative ultrasound comparison. Each patient first received a B-mode transvaginal ultrasound examination and the cervical tumoral tissue was identified as a mass with heterogeneous echogenicity and irregular borders with disruption of the cervical canal. The optimal gray-scale image of the sagittal view along the longest diameter of the cancer was obtained. B-mode scanning was followed by dual-mode scanning for real-time elastography with color-encoded superimposition of the information. Elastographic images were generated by soft and rhythmic compression of the cervix using the ultrasound transducer. On the monitor, the two panel image was displayed with the conventional B-mode image on the left and the elastography image on the right. The elasticity information is presented in color, with blue indicating stiffer tissue, red indicating softer tissue and green as intermediate stiffness.

All elastography images were analyzed by 2 experienced ultrasonographers (X.X., X.X.X.)with 12 and 15 years’ experiences in gynecology. Strain ratio was employed to evaluate the strain difference between the cervical cancer and the normal parametrial tissue quantitatively. A press indicator on the left upper side of screen was used to evaluate the condition of compression in the region of interest (ROI) from the minimum to the maximum (level 1–6). Compression and relaxation waveforms were shown on the lower right side screen. While the compression indicator was green color at the value of 5 or 6 and the pressure waveform was simultaneously at the peak, the images of measurements and examination techniques were stored digitally. To compute the strain ratio, we first circled the normal parametrial tissue at the same depth of the cervical cancer as A,then manually contoured entire cervical cancer as B, and strain ratio was computed as A/B. We performed this three times for each patient and computed the mean value of strain ratio.

### Statistical analysis

The clinical characteristics of the patients and tumors were expressed in mean and standard deviation (SD). Mann-Whitney test was used to compare the difference in FIGO stage, histological grade and lymphatic metastasis between the CR and PR groups. The comparison of mean age and the maximum tumor diameter between two groups were performed using Student’s unpaired t-test. Repeated measures analysis of variance (ANOVA) and Student’s unpaired t-test were constructed to the multiple comparisons in strain ratios for the CR group and PR group at each time point. A two-sided *p* < 0.05 was considered to be statistically significant. The inter-observer and intra-observer variability of measurements were assessed using intra-class correlation coefficients (ICCs) with a 95% confidence internal (CI). Statistical analysis was performed using SPSS software version 19.0 (SPSS Inc., Chicago, IL,USA).

## Results

### Patient and tumor characteristics

Of the 47 enrolled patients, 36 patients were included in this analysis. The remaining 11 patients were excluded from the study due to incomplete follow-up imaging study or clinical evaluations. The tumor characteristics are summarized in Table 1. From the MRI evaluations, 25 (69.4%) patients were classified as CR, 11 (30.6%) patients as PR, no patients as SD or PD. The age range was 34–77 for the CR group and 31–67 for the PR group (*p* = 0.131). Prior to treatment, the mean maximum tumor length was 35 ± 15 mm for the CR groupand 42 ± 14 mm for the PR group (*p* = 0.193). There was no significant difference between the CR and PR groups in FIGO stage (*p* = 0.453), histological grade (*p* = 0.359) or lymphatic metastasis (*p* = 0.621).

**Table 1:** Tumor Characteristics

Characteristics	No. of Patients (*n* = 36)
*FIGO stage*	
IB	2 (5.6%)
IIA	4 (11.1%)
IIB	18 (50.0%)
IIIA	3 (8.3%)
IIIB	6 (16.7%)
IV	3 (8.3%)
*Histological grade*	
High	6 (16.7%)
Intermediate	26 (72.2%)
Poor	4 (11.1%)
*Histological type*	
Squamous cell carcinoma	36 (100%)
*Lymphatic Metastasis*	
Yes	24 (66.7%)
No	12 (33.3%)

*FIGO* the International Federation of Gynecology and Obstetrics

### Ultrasound elastography assessment of treatment response

**Fig. 1:**
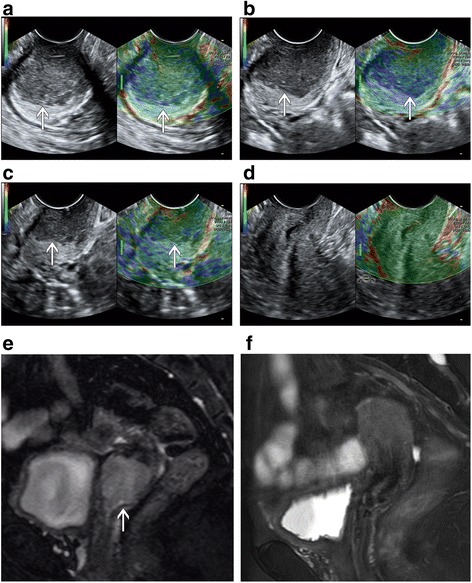
A patient with advanced cervical cancer (FIGO stage IIB) experienced complete response to concurrent chemo-radiotherapy(CCRT). B-mode and elastography images show a significant decrease in strain ratio in cervical cancer (arrow): 4.17 prior to CCRT **a** 3.03 at week 1 during CCRT **b** 2.73 at week 2 during CCRT **c** and 1.4 post CCRT **d** Corresponding axial T2-weighted images exhibited a significant decrease in the maximal diameter of tumor (arrows): 3.5 cm at pre-therapy **e** and 0 cm post therapy **f**

**Fig. 2:**
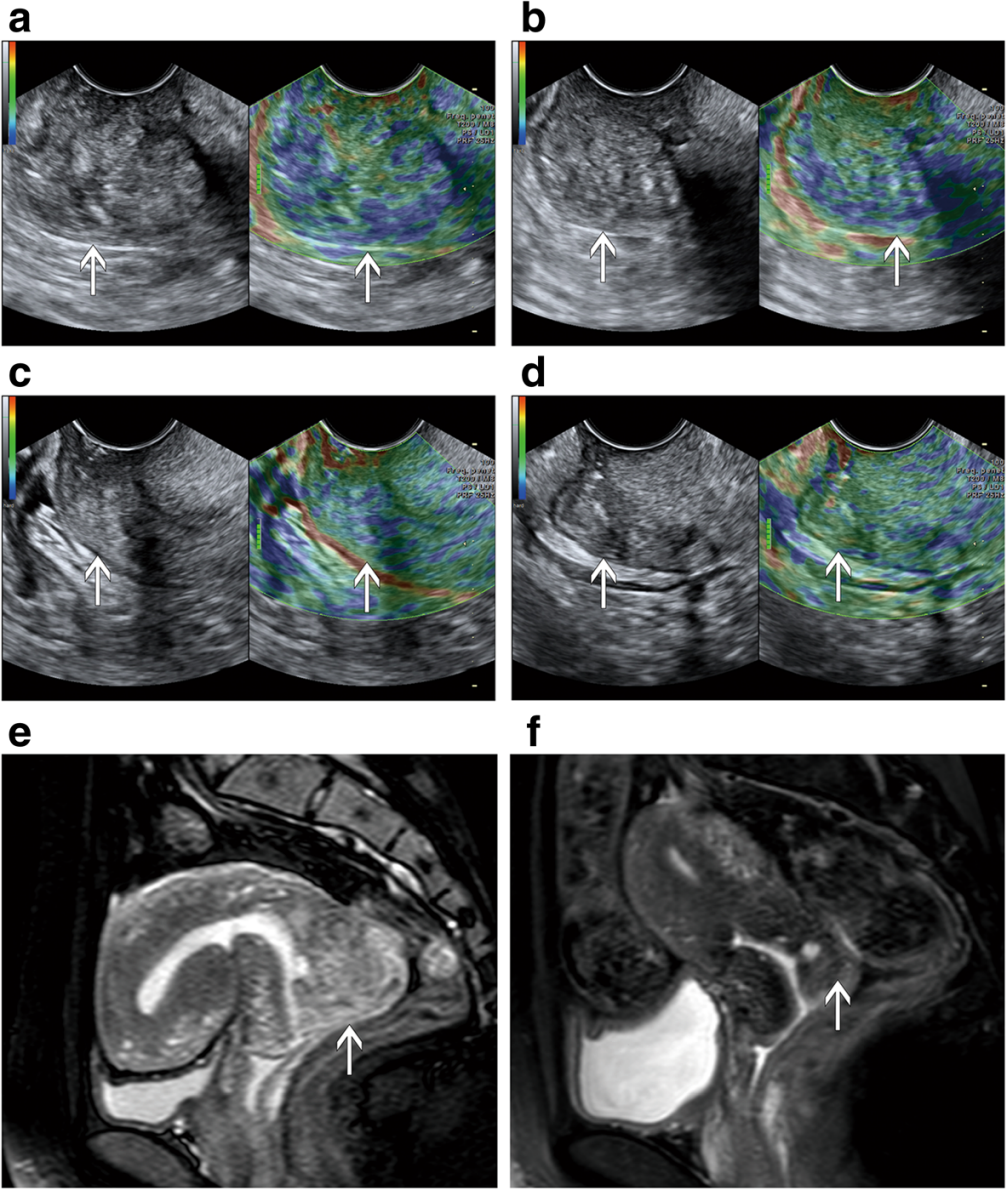
A patient with advanced cervical cancer (FIGO stage IIA) experienced partial response to CCRT. B-mode and elastography images show a consecutive decrease in strain ratio in cervical cancer (arrow): 4.16 before therapy **a** 3.56 at week 1 during CCRT **b** 3.07 at week 2 during CCRT **c** and 2.73 post CCRT **d** Corresponding axial T2-weighted images exhibited a decrease in the maximal diameter of tumor (arrows): 3.3 cm at pre-therapy **e** and 1.2 cm post therapy **f**

**Fig. 3:**
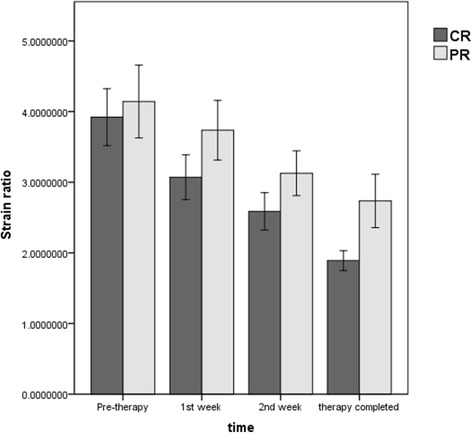
Dynamic changes of mean strain ratios of cervical cancers during concurrent chemo-radiotherapy (CCRT) in the complete responders (CR) and partial responders (PR). The strain ratios were decreased consecutively in CR and PR groups during the treatment course and significant differences were found in strain ratios between CR and PR group from week 1 during CCRT to therapy completion (all *p* < 0.05)

Figure 1 showed the B-mode and elastography images of a representative complete responder case at 4 time points and corresponding axial T2-weighted images prior to and right after therapy completion, while Fig. 2 illustrated a partial responder case. Table 2 summarizes the mean strain ratios of the tumors in the complete and partial responders at each time point, and Additional file 1 (Figures S1 and S2) displayed the mean strain ratios of each patient in CR and PR groups. Before starting treatment, CR group and PR group demonstrated similar tumor stiffness, with average strain ratios of 3.92 ± 0.98 and 4.14 ± 0.77, respectively. The strain ratios were significantly different between time points in the CR group (*F* = 87.004, *p* < 0.001) and PR group (*F* = 38.317, *p* < 0.001), and the difference between CR and PR groups was found to be significant (*F* = 7.203, *p* = 0.011). Strain ratios between CR group and PR group were significant from 1 week after treatment initiation to therapy completion (all *p* < 0.05). Compared to strain ratios at pre-therapy (baseline), Fig. 3 exhibited that significant decreases in CR group were seen from 1 week after treatment initiation to therapy completion (all *p* < 0.001), however, PR group showed significant differences from 2 weeks to therapy completion (*p* = 0.001, *p* < 0.001, respectively), but no statistical significance at 1 week (*p* = 0.084).

**Table 2:** The mean strain ratios of the tumors in the complete and partial responders at each time point

Group	Time points				Sum	*F*	*P*
	Pre Tx	Post T1	Post T2	Post T3			
CR	3.92 ± 0.98	3.07 ± 0.77	2.59 ± 0.64	1.89 ± 0.34	2.87 ± 1.03	87.004	< 0.001
PR	4.14 ± 0.77	3.74 ± 0.63	3.13 ± 0.47	2.74 ± 0.56	3.42 ± 0.82	38.317	< 0.001
sum	3.99 ± 0.91	3.27 ± 0.78	2.75 ± 0.64	2.15 ± 0.57	3.04 ± 1.00^a^	91.723^a^	< 0.001^a^
t	0.664	2. 515	2.503	5.583	7.203^a^	(*F* = 2.922, *P* = 0.058)^#^	
P	0.511	0.017	0.017	< 0.001	0.011^a^		

*CR* complete responder, *PR* partial responder, *Pre Tx* pre-therapy (baseline), *Post T1* at 1 week during CCRT, *Post T2* at 2 weeks during CCRT, *Post T3* CCRT completion (within 1 week)

^a^F statistic and *P* value of main effect; ^#^F statistic and *P* value of crossover effect

The ICC between two observers was 0.986 (95% CI 0.947–0.996; *p* < 0.001), and the intra-observer variability was 0.991 (95% CI 0.964–0.998; *p* < 0.001).

## Discussion

This study provided initial evidence that strain elastography may be used to monitor and predict therapeutic response of CCRT in patients with cervical cancer. It is well-known that tumor formation and its degeneration in response to CCRT may exhibit corresponding changes such as inflammation and fibrosis with stromal cells [25, 26], which substantially alter the biomechanical properties of tumor tissues. After therapy completion, all patients were classed by MRI as complete or partial responders. The cervical cancer appeared a decrease in the strain ratio, thus indicating an increase in the tumor strain and a decrease in stiffness. The strain ratios revealed significant changes between time points in the CR group (*F* = 87.004, *p* < 0.001) and PR group (*F* = 38.317 *p* < 0.001), and the difference between CR and PR groups showed significant (*F* = 7.203, *p* = 0.011). Strain ratios between CR group and PR group were significant from week 1 during CCRT to therapy completion (all *p* < 0.05). Moreover, strain ratio exhibited a significant decrease at week 1 during CCRT for the CR group (*p* < 0.001) and at week 2 for the PR group (*p* = 0.001).

In our study, the strain ratio of the pre-treatment cervical cancer (mean 3.99) is in line with the published results (mean 3.8) and significantly different from normal cervical tissues (mean 1.2) [22]. The high strain ratio of cervical cancer is due to the increased cellular proliferation, which is an important factor that influences the strain in tumor tissue. In a study of 55 breast-cancer patients, Hayashi et al. [27] investigated the tumor stiffness post neoadjuvant chemotherapy and found that relatively soft tumors were highly responsive to neoadjuvant chemotherapy and more frequently displayed pathologic complete response as compared with hard tumors. However, in our study, the baseline strain ratios were not significantly different between the CR and PR groups, which were consistent with the study reported by Rafaelsen et al. [20] and the reason may be relevant to the identical histological type. Additionally, there were no significant differences in the age, tumor size, FIGO stage, histological grades, and lymphatic metastasis between these two groups. Therefore, the therapy response doesn’t seem to correlate with the baseline strain ratio, tumor size, patient age, FIGO stage, histological grades, or lymphatic metastasis.

Due to tumor heterogeneity of radioresponsiveness, the timing of optimal evaluation for therapeutic response to CCRT remains controversial. Thus, how to identify patients at risk of treatment failure in the early stage is very important for clinicians. Recently, several studies have focused on the early detection of response to chemoradiation in cervical cancer using DW and DCE-MRI. Harry et al. [28] reported that tumor apparent diffusion coefficients (ADCs) after 2 weeks of CCRT were significantly correlated with final tumor response, while Liu et al. [29] conflicted with this results, showing significant correlation with final therapeutic responses at 4 weeks after initiation of CCRT. Moreover, a recent study of Park et al. [30] demonstrated that the mean ADC and tumor volume transfer constant K(trans) and extravascular extracellular volume fraction (ve) of cervical cancer increased even 1 week after initiating CCRT. To date, the role of sonoelastography as a predictor of therapeutic response has been demonstrated in some tumors. Rafaelsen et al. showed elastography after two weeks of chemo-radiation seems to hold early predictive treatment response information for rectal cancers [20]. However, a study conducted by Falou et al. [31] using ultrasound elastography suggested that there was no significance at 1 week after the start of neoadjuvant chemotherapy with breast cancer patients, while non-responders and responders were found to be highly significantly different 4 weeks after treatment initiation for averaged strain ratios. In our series, strain ratios for CR group and PR group were significant at 1 week after treatment initiation (*p* < 0.05). Moreover,strain ratios showed a significant decrease after 1 week of CCRT in CR group. These results might be explained that with the effective treatment, the tumor begins to become less stiff as a result of a decrease in cellular proliferation and moderate induction of apoptosis which changes its structure and biomechanical properties, leading to an increase of tumor strain and a decrease of strain ratio. Taken in aggregate, these findings suggest that the potential of strain elastography as a surrogate biomarker to evaluate an early therapeutic response.

With successful therapy, treatment responses are tumor cell necrosis, apoptosis and lysis, resulting in a decrease in tumor stiffness. After therapy completion, our results showed that strain ratios of CR and PR groups both significantly decreased (both *p* < 0.001), which were strongly correlated with clinical assessment. These findings pave the way for clinical application of strain elastography imaging in monitoring CCRT of cervical cancer. It is clear that if any quantitative features are used to assess tumor response, reproducibility is a prerequisite. Our study further demonstrated the inter-observer and intra-observer reliability of strain ratio evaluations, which were similar to the findings reported by Sun et al. [18]. Future studies will be designed to further investigate the correlation between change in stiffness and treatment response in non-responders.

Tumor size is one of the most important prognostic factors for cervical cancers [32]. With the superior soft tissue contrast resolution, MRI displays high accuracy (70%) for determination of tumor size [33] and has been considered as the reliable method strongly recommended by RECIST 1.1 for follow-up in tumor size delineation after non-surgery treatment [24]. Vincens et al. demonstrated that the sensitivity of MRI in evaluating residual tumor were 80% in stage IB2/II cervical carcinoma after CCRT [34]. A prospective study on cervical cancer showed that tumor regression rate obtained during mid-radiation therapy had the best outcome prediction rate for local control (84%) and disease-free survival (63%) [35]. In this study, as ultrasound was not recommended to measure tumor lesions during therapy [24], tumor sizes were measured by MRI.

Several limitations exist in this study. First, this study was performed with small sample size and a short follow-up period. Second, our study observed morphologic changes before and after treatment based on imaging by MRI as a reference of therapeutic response and lacked pathologic results and clinical outcome, such as progression-free survival and overall survival. Third, we did not investigate the effect of CCRT on the stiffness of normal parametrial tissue in the current study. Fourth, only cervical squamous carcinoma was included in this study, so we are uncertain whether other types of cervical cancers will show similar trends in the treatment response monitoring by strain elastography.

## Conclusions

This study demonstrates the feasibility of using strain elastography imaging to monitor the treatment response of cervical cancer during CCRT. Furthermore, the significant decrease in strain ratios after 1 week of treatment for the CR group indicates its potential role as an early predictor of treatment response. These findings warrant future clinical studies to refine the strain elastography technique with the ultimate goal to provide reliable imaging biomarkers to adjust ineffective therapy promptly and optimize personalized therapy.

## Additional file

Additional file 1: Figure S1. The mean strain ratios of each patient with advanced cervical cancer experienced complete response to CCRT at four times, respectively: a) before therapy, b) at week 1 during CCRT, c) at week 2 during CCRT, d) post CCRT. **Figure S2.** The mean strain ratios of each patient with advanced cervical cancer experienced complete response to CCRT at four times, respectively: a) before therapy, b) at week 1 during CCRT, c) at week 2 during CCRT, d) post CCRT. (RAR 3131 kb)

## Abbreviations

ADC: Apparent diffusion coefficient
ANOVA: Repeated measures analysis of variance
CCRT: Concurrent chemo-radiotherapy
CI: Confidence internal
CR: Complete response
DCE: Dynamic contrast-enhanced
DW: Diffusion-weighted
EBRT: External beam radiotherapy
FDG-PET: Fluorodeoxyglucose-positron emission tomography
FIGO: International federation of gynecology and obstetrics
ICC: Intraclass correlation coefficients
ICR: Intracavitary brachytherapy
MRI: Magnetic resonance imaging
PD: Progress disease
PR: Partial response
RECIST: Response Evaluation Criteria in Solid Tumors
ROI: Region of interest
SD: Stable disease
SD: Standard deviation.
